# An Update on Laboratory Diagnosis of Liver Inherited Diseases

**DOI:** 10.1155/2013/697940

**Published:** 2013-10-08

**Authors:** Federica Zarrilli, Ausilia Elce, Manuela Scorza, Sonia Giordano, Felice Amato, Giuseppe Castaldo

**Affiliations:** ^1^Dipartimento di Bioscienze e Territorio, Università del Molise, Isernia, Italy; ^2^CEINGE-Biotecnologie Avanzate Scarl, Via Gaetano Salvatore 486, 80145 Naples, Italy; ^3^Università Telematica Pegaso, Naples, Italy; ^4^Dipartimento di Medicina Molecolare e Biotecnologie Mediche, Università di Napoli Federico II, Naples, Italy

## Abstract

Liver inherited diseases are a group of genetically determined clinical entities that appear with an early chronic liver involvement. They include Wilson's disease (hepatolenticular degeneration), hereditary hemochromatosis, and alpha-1-antitrypsin deficiency. In addition, cystic fibrosis, although it is not specifically a liver disease, may cause a severe liver involvement in a significant percentage of cases. For all these pathologies, the disease gene is known, and molecular analysis may contribute to the unequivocal diagnosis. This approach could avoid the patient invasive procedures and limit complications associated with a delay in diagnosis. We review liver inherited diseases on the basis of the genetic defect, focusing on the contribution of molecular analysis in the multistep diagnostic workup.

## 1. Introduction

Although a chronic liver involvement may be observed in a series of genetic diseases, three of them are typically reported as inherited liver diseases because liver may be the principal target organ, that is, Wilson's disease (hepatolenticular degeneration), hereditary hemochromatosis (HH), and alpha-1-antitrypsin (AAT) deficiency [1]. In addition, liver disease with a different severity may be present in up to one-third of patients with cystic fibrosis (CF), the most frequent autosomal recessive fatal disorder among Caucasians. For these reasons we will include CF in the present review. The penetrance of liver expression in such diseases is widely different, since the liver damage is modulated by the genetic background of each patient [2]. We critically discuss the most recent advances in the pathogenesis of such disorders, with particular regard to biochemical and molecular approaches that in the last decade permitted earlier and more specific diagnoses, reducing the need of invasive bioptic approaches [3].

## 2. Wilson's Disease (Hepatolenticular Degeneration)

### 2.1. Introduction and Epidemiology

Wilson's disease (WD) was described in the Ph.D. thesis by S. Wilson in 1912 as progressive brain lenticular degeneration with cirrhosis. It is an autosomal recessive disorder with an incidence of 1 : 30,000 newborns. WD typically includes liver disease that appears in the second decade followed by neurological disorders in the third decade. Severe cases with earlier onset and mild cases with a later onset have been described [4]. The identification of the disease gene greatly improved the diagnosis of WD, and some experience of newborn screening was performed [5]. Novel therapies can now effectively treat patients bearing such disease once fatal, and gene therapy is effective in animal models [6, 7].

### 2.2. Liver Disease in Hepatolenticular Degeneration

Liver disease in WD patients is widely variable ranging from asymptomatic cases with mild hepatomegaly and a slight serum increase of hepatocellular enzymes, to chronic liver disease with severe steatosis, up to fibrosis, cirrhosis, and severe liver failure [8]. In about two-thirds of cases there is haemolytic anemia, coagulopathy, and renal failure (mainly due to tubular damage). A percentage of patients with acute liver onset (acute/fulminant liver failure with or without haemolysis) have been described that require a rapid diagnosis and treatment [9]. Also neurological expression is heterogeneous in WD patients and is dominated by motoric dysfunction (postural tremor and intention tremor and in one-third of cases, tremor of the trunk and head). About a half of patients have psychiatric disturbances, mainly depression up to psychotic symptoms. Interestingly, such alterations may be reverted by adequate therapy [10]. 

### 2.3. Genetics and Pathogenesis

Wilson's disease is due to mutations in the gene encoding the ATP7B Cu translocase (copper-transporting P-type ATPase). The protein is mainly expressed by the hepatocyte and regulates the levels of copper in the liver (thus, in the organism, because most dietary copper is present in hepatocytes). Furthermore, ATP7B modulates the synthesis of ceruloplasmin [11]. In particular, copper hepatocyte levels regulate the topogenesis of the ATP7B protein: if copper levels are normal, the ATP7B protein is retained in Golgi and contributes to regulate the synthesis of cuproproteins and to distribute copper among intracellular newly produced cuproproteins. Once intracellular levels of copper increase, the ATP7B protein is mainly addressed to the canalicular membrane and helps to excrete copper through the bile (that represents the excretion mechanism for more than 98% of copper from the body). When the activity of ATP7B at membrane level is impaired, as it occurs in WD patients, copper accumulates within the hepatocyte. In its free form, it has a relevant redox potential and induces oxidative stress attacking cell membranes, proteins, and DNA particularly at liver level (where most copper of the body is present) and in the brain. The pathogenesis of brain damage is not well known. It involves neurons of the extrapyramidal system with inflammation and irreversible impairment [12].

About 300 different mutations have been described so far. Studies of genotype-phenotype correlation concluded that severe mutations (i.e., nonsense, frameshift) are typically associated with the most severe disease, while for patients bearing missense mutations (about 60% of all mutations), it is more difficult to predict the expression of the disease [4]. 

### 2.4. Diagnosis

The diagnosis of WD is difficult to perform because either liver or neurological alterations are aspecific (although the coexistence of liver and neurological symptoms may be suggestive for WD). Kayser-Fleischer rings are present in about 50% of cases of WD at diagnosis. However, they may be present also in patients with chronic cholestasis, primary biliary cirrhosis, and cryptogenetic cirrhosis. The presence of low serum ceruloplasmin and higher urine copper is sufficient to conclude the diagnosis of WD in most cases, even if a percentage of patients may have normal levels of such markers [7, 13]. Liver biopsy, necessary to perform the diagnosis before molecular analysis [3], is now used to define liver status in cases with ambiguous biochemical parameters and to evaluate hepatic copper levels with specific stains. Molecular analysis is available [14, 15]; there are some more frequent mutations, but given the strong genetic heterogeneity of WD, the scanning of the gene (by sequencing) is required, reaching a detection rate of about 95%. However, gene sequencing often reveals novel mutations for which it may be difficult to define the pathogenic effect.

## 3. Hereditary Hemochromatosis

### 3.1. Introduction and Epidemiology

Hereditary hemochromatosis (HH) is a late onset autosomal diseases characterized by enhanced iron intestinal absorption and iron overload that may lead to liver cirrhosis, cardiomyopathy, diabetes, arthritis, and skin pigmentation. Most of such alterations are not peculiar to HH, thus rendering complex the differential diagnosis. Furthermore, clinical symptoms appear late during the history of HH, and finally a series of diseases may cause secondary hemochromatosis. For all these reasons HH is often undiagnosed, and its incidence is underestimated. This is a relevant problem, considering that hemochromatosis is not treated among the major risk factors for hepatocellular carcinoma [16].

The incidence of HH depends on the diagnostic approach used. Surprisingly, if the diagnosis is based on traditional laboratory markers (i.e., serum ferritin and transferrin saturation) the incidence of the disease is higher as compared to that resulting from the analysis of hemochromatosis gene (*HFE*) mutations [17]. However, an incidence of 1 : 250 individuals (corresponding to a prevalence of 1.6–5.9 for 1,000 inhabitants) is commonly accepted, rendering HH one of the most common genetic disorders in Caucasians. The incidence of HH is higher in northern Europe (i.e., Ireland), and there is a gradient of incidence between northern and southern Europe.

### 3.2. Liver Disease in Hereditary Hemochromatosis

While in secondary hemochromatosis the iron overload typically involves macrophages, in HH iron mainly accumulates at hepatocellular level triggering a chronic liver damage that ends in hepatic fibrosis and cirrhosis [18], with a number of patients that evolve to hepatocellular carcinoma [16]. The pathogenesis of liver damage in HH is mainly related to the oxidative damage induced by iron followed by a rapid lipid peroxidation of mitochondria, microsome, and lysosome membranes. Kupffer cells react and actively release cytokines that in turn stimulate stellate cells to produce collagen, thereby leading to fibrosis [18]. 

Signs and symptoms of hemochromatosis depend on the phase of the disease. When HH is diagnosed through screening (or by occasional laboratory evaluation), most patients are asymptomatic, because tissue and organ damage typically appear after 30 years of age. If the diagnosis is performed for symptoms, HH may appear with abdominal pain, arthralgias (usually involving metacarpophalangeal joints), chondrocalcinosis, and altered libido and symptoms like heart failure, diabetes, and liver insufficiency. Liver disease in HH includes hepato- and splenomegaly in the initial phase (with altered serum hepatocellular enzymes), followed by the gradual reduced protidosynthetic activity (ascites, portal hypertension, and encephalopathy). Of course, liver damage may be further worsened by concurrent alcoholic chronic intake, viruses, or other diseases [18].

### 3.3. Genetics and Pathogenesis

 Hereditary hemochromatosis is frequently associated with the *HFE* p.C282Y homozygous genotype particularly in northern Europe. However, only a percentage of such subjects develop an overt hemochromatosis requiring therapy, and recently, in southern France a registry was created to evaluate the prevalence of symptomatic p.C282Y homozygous subjects [19]. On the other hand, HH may be present in subjects negative to *HFE* gene mutations, particularly in geographic area different from northern Europe [20]. The interesting correlations between hemochromatosis and celiac disease: a first study demonstrated that occult celiac disease prevents penetrance of hemochromatosis [21]; later, it was evidenced that the C282Y mutation of *HFE* gene may mitigate the severity of celiac disease [22], and finally a large case-control study concluded that hemochromatosis predispose to celiac disease [23]. 

 In the last years the iron homeostasis was studied either in normal subjects or in patients with hemochromatosis, revealing that several proteins, encoded by at least 5 genes may be altered in HH patients [24] causing forms of hereditary hemochromatosis with a different severity and age at onset (Table 1). In synthesis, ferroportin (encoded by *SLC40A1*, mutated in type 4 HH) modulates the iron efflux by macrophages and enterocytes. Hepcidin (encoded by *HAMP*, mutated in juvenile hemochromatosis, i.e., type 2B) binds and induces the degradation of ferroportin; the absence (or the reduced activity of hepcidin) causes a higher activity of ferroportin and thus a higher iron absorption. HFE (altered in Type 1 HH), HJV (altered in Type 2 A HH), and TFR2 (altered in Type 3 HH) regulates the synthesis/activity of hepcidin. Thus, their absence or reduced activity causes the reduced synthesis of hepcidin and thus a higher activity of ferroportin.

### 3.4. Diagnosis

 The diagnosis of hemochromatosis is mainly based on (i) enhanced serum ferritin levels (>300 microg/L in men and >200 micog/L in females), which correlates with the increased iron content of liver and (ii) the high transferrin saturation (>50% in males and >45% in females). Other studies suggest that transferrin saturation is the preferred screening test, but it is important that each laboratory collects its reference values for iron biochemical markers, given the strong racial variability [25]. Unsaturated iron-binding capacity has the same (or higher) diagnostic performance of transferrin saturation [26] at lower cost, but its use for screening is limited by the higher biological variability [25]. In addition to diagnosis, laboratory has a role in the monitoring of patients in the different phases of the natural history of HH through biochemical markers of (i) liver fibrosis [27]; (ii) liver protidosynthetic [28]; (iii) hepatocarcinoma in patients with cirrhosis [29].

Molecular analysis would confirm hereditary hemochromatosis, but it has a variable diagnostic sensitivity because the mutations are different in different geographic areas. Thus, in patients with altered ferritin and transferrin saturation and negative molecular analysis, all causes of secondary hemochromatosis are to be excluded [18], among which iron-loading anemias and viral and alcoholic chronic liver diseases. Different protocols for molecular analysis have been suggested: (i) the analysis of *HFE* p.C282T mutation alone or in combination with the p.H63A variant as first step, followed by the analysis of rare variants in negative patients with positive markers of iron overload with no other explanation [30]; (ii) the analysis of *HFE* p.C282T alone, being the homozygous presence of such mutation the lone genotype that confirms the diagnosis of HH, according to the French Health Authority [19]; the compound heterozygosity of the p.C282T with the p.H63A or with the p.S65C may be found in patients with mild HH [19]; (iii) the scanning analysis of *HFE* gene, given the high genetic heterogeneity of HH; of course such approach would increase the detection rate of molecular analysis, but it would often identify novel mutations for which it may be difficult to define the pathogenic effect [31]; (iv) the analysis of *HFE* p.C282T and H63A mutations followed by the analysis of mutations in other putative disease genes for HH like hemojuvelin (the G320V mutation is the most frequent mutation found in juvenile HH), hepcidin, transferrin receptor 2, and ferroportin [32]. In fact, unlike European countries, in Asia-Pacific regions only a small percentage of HH is due to the p.C282Y *HFE* mutation [20]. Finally, in some geographic areas mass screening programs for HH based on molecular analysis of the C282Y mutation or on molecular analysis and biochemical marker of iron overload have been performed, in order to prevent disease evolution [33]. 

Liver biopsy was fundamental to perform diagnosis before the availability of molecular analysis [3]. Currently, it is performed in strongly suspected patients with negative genetics and ambiguous biochemical markers; in addition, it may be used to assess the degree of liver fibrosis and cirrhosis and the degree of iron liver overload [34]. However, noninvasive approaches to assess hepatic fibrosis based on biochemical markers or on MRI elastography [27, 35] are currently under study. Finally, magnetic resonance imaging has a role in quantification of liver iron [36]. 

## 4. Alpha-1 Antitrypsin Deficiency

### 4.1. Introduction and Epidemiology

Alpha-1 antitrypsin deficiency is an autosomal codominant disease with variable penetrance and expressivity that affect the lung and the liver. The disorder is caused by mutations in the *SERPINA1* gene encoding for alpha-1-antitrypsin glycoprotein, a member of serine protease inhibitor superfamily (serpins) and produced mainly by the liver. The primary role of AAT is inhibition of neutrophil elastase, an inflammatory proteinase. The most important physiological functions of AAT are the protection of lung tissue by proteolytic enzymes and the regulation of lung immune processes [37].

The clinical expression of AAT deficiency consists in chronic lung disease, (mainly of pulmonary emphysema sometimes associated with disseminated bronchiectasis or, more rarely, asthma) between the 4th and the 5th decade of life [38], and/or a liver pathology, which may occur within the 1st year of life or later, up to the 5th and 6th decade, in the form of chronic liver disease, cirrhosis, until hepatocellular carcinoma [39]. Other clinical phenotypes rarely associated with the alpha-1 antitrypsin deficiency are necrotizing panniculitis and vasculitis [40]. 

Alpha-1 antitrypsin deficiency has an incidence of 1 : 2000–5000 newborn. The incidence is higher in Northern Europe and America and tends to decrease according to a north-south gradient. Overall, the prevalence of the disease in the populations of western Europe is about 1 : 2500 and is closely related to the Scandinavian descent in the population. In all countries, however, the number of patients clinically identified is far lower than the prevalence based on the allele frequencies. 

### 4.2. Liver Disease in Alpha-1 Antitrypsin Deficiency

The presentation of AAT deficiency-associated liver disease is highly variable and ranges from chronic hepatitis and cirrhosis to fulminant hepatic failure. The underlying cause may be the intrahepatic accumulation of polymerized alpha-1 antitrypsin molecules. Subsequent intrahepatic cholestasis may lead to a reduced resorption of lipids and lipophilic vitamins. The progression to cirrhosis in patients with AAT deficiency is slow. However, some patients develop early end-stage disease, with the need for liver transplantation. The incidence of primary liver cell carcinoma is higher than in other chronic hepatic diseases.

The typical presentation in the neonatal period is cholestasis, abdominal distention, pruritus, poor feeding, poor weight gain, hepatomegaly, and splenomegaly. Many children appear to be completely healthy, without evidence of liver injury. Clinical signs in newborns are not necessarily associated with liver disease in adulthood. 

Liver disease in adults appears with chronic hepatitis, with or without cirrhosis. Nevertheless, adults with alpha-1 antitrypsin deficiency-associated genotypes develop liver disease less frequently than pulmonary manifestations. Monitoring for progression of the liver disease is necessary to transfer the patient to a liver transplant centre. AAT replacement therapy has no effect on the liver disease since liver injury is related to the accumulation of the AAT mutant protein within hepatocytes and not to the lack of circulating antiprotease activity [41].

### 4.3. Genetics and Pathogenesis

*SERPINA1* gene is highly polymorphic, and to date more than 100 allelic variants have been identified. Such variants can be classified according to their effect on serum levels of alpha-1-antitrypsin. The alleles M (M1 to M6) are the most common and are defined as “normal alleles" because they are associated with normal serum levels of AAT (20–53 mmol/L). Most patients with liver or lung involvement are homozygous for the Z or the S allele or compound heterozygous for the 2 alleles. In these patients serum levels of AAT are reduced by 40–60% compared to normal. The most common form of AAT deficiency occurs in homozygous for the Z allele in which the serum concentration of AAT is about 3.5–7 mmol/L or approximately 15% of normal values. Most patients with ZZ or SZ genotype, and some with SS genotype, have lung or liver disease. Heterozygous individuals (MZ or MS) rarely develop clinical signs of disease. Finally, up to 60 variants are known silent and a few, rare variants, may have a pathogenic role [42]. In Italy, such rare variants are responsible for 11% of cases of severe deficiency of alpha-1 antitrypsin. In addition, the penetrance of the *SERPINA1* gene is relatively poor: it is estimated that less than 30% of subjects affected by severe deficiency of alpha-1-antitrypsin have a clinical expression of the disease.

### 4.4. Diagnosis

Alpha-1 antitrypsin deficiency is significantly underdiagnosed, and the diagnosis is often delayed for several years. An algorithm for the diagnosis of AAT diagnosis is not yet well defined. However, the laboratory diagnosis includes the determination of serum levels of AAT and a quantitative determination of AAT through the isoelectric focusing (IEF) and genotyping. The determination of serum levels of AAT in subjects without inflammatory diseases is the best initial test to identify the severe AAT deficiency. In fact, since the AAT is an acute phase protein, systemic inflammation induces the protein expression, raising its serum levels [42]. Thus, even during the exacerbation of pulmonary inflammatory disease, levels may be higher than the stable phase. It is recommended that CRP levels are measured simultaneously to AAT and that the results of the concentration of AAT are not considered if CRP levels are pathological. In addition, the immunoelectrophoretic analysis of the Z heterozygotes may have false negative results. 

The indications for the determination of serum concentration of AAT include [43] early onset of emphysema or emphysema; liver disease not otherwise explained; necrotizing panniculitis; antiproteinase 3 positive vasculitis; bronchiectasis; family history of emphysema, bronchiectasis, panniculitis, and liver disease.

In addition to the index case, the relatives should be analyzed to identify asymptomatic carriers. Finally, all infants with prolonged jaundice or nonspecific signs of liver disease should be tested for AAT deficiency. Genotype analysis is able to detect or exclude *SERPINA1*-specific mutations in the gene coding for the alpha-1-antitrypsin: the results are reliable but limited to the most common variants. In fact, also sequencing the whole gene, when a rare variant is identified, it is difficult to define its pathogenetic role. Furthermore, the expression of alpha-1 antitrypsin deficiency is variable even within the same family or in carriers of the same molecular defects; thus, patients must be counselled about the significance of molecular data that genetic testing offers.

Genetic counselling is very useful for explaining in detail the meaning of the possible genotypes that can be identified and the usefulness of the knowledge of its own variant regarding the procreative risk. The identification of the molecular defect will enable extending the molecular investigation to family members at risk in order to early intervene if asymptomatic carriers are positive. 

When a patient is identified as a new case of AAT deficiency for Z allele homozygosity, it is crucial to establish the transmission for the descendants. Prenatal diagnosis is not a routine procedure for the low penetrance of liver disease shortly after birth. However, it is a crucial step in the cases in which parents, after typing of the alpha-1 antitrypsin locus, are carriers for the Z allele or null allele.

## 5. Cystic Fibrosis

### 5.1. Introduction and Epidemiology

Cystic fibrosis (CF) is the most frequent (1 : 2,500 newborn) lethal inherited disease among Caucasians. It typically appears with pancreatic insufficiency and pulmonary disease due to a vicious circle of inflammation and bacterial colonisation that gradually lead to respiratory insufficiency [44]. Clinical forms showing pancreatic sufficiency, single organ involvement, and a less severe outcome are included under the umbrella term of *cystic fibrosis transmembrane regulator*-related disorders (CFTR-RD). This term includes clinical entities associated with *CFTR* dysfunction that does not fullfill diagnostic criteria for CF such as congenital bilateral absence of the vas deferens (CBAVD), chronic pancreatitis, and disseminated bronchiectasis [45]. 

### 5.2. Liver Disease in Cystic Fibrosis

Liver disease in CF patients depends on the altered activity of *CFTR* chloride channel on the apical membrane of cholangiocytes. This altered activity/amount causes a defective (sluggish) bile flow and is associated with a cholangiocyte-induced inflammatory response with proliferation of stellate cells, which gives rise to cholangitis with biliary obstruction and progressive periportal fibrosis [46]. CF-associated liver disease is typically slowly progressive, but in up to 10% of children may rapidly evolve to multilobular biliary cirrhosis and portal hypertension. In these patients liver involvement may be the major manifestation of CF. However, portal hypertension and gastrointestinal haemorrages in CF are usually associated with a maintained hepatocellular activity. It means that the treatment of varices effectively reduces the risk of mortality for liver CF [47]. 

It is still unknown why only a subgroup of CF patients develops liver disease and the causes of its different severity [47]. It would be useful to predict liver expression in CF patients, given the potential of ursodeoxycholic acid therapy in the early stages of CF liver disease [46] and the opportunity of liver transplantation once pulmonary function is well preserved. 

The prevalence of liver involvement in CF patients is estimated around one-third of cases. However, it varies depending on the diagnostic criteria [47]. CF patients show postmortem alterations ranging from focal biliary cirrhosis to multilobular cirrhosis in up to 70% of cases, but less than 10% of CF children display a significant liver involvement with portal hypertension. The pathogenesis of liver expression is poorly known. The weak correlation between the *CFTR* genotype and liver expression in CF patients emerged from studies on patients bearing F508del (the most frequent disease-causing mutation) and expressing a discordant liver phenotype. Also our group described a CF patient homozygous for the G542X severe mutation who had a very severe liver phenotype, unlike the six previously reported CF cases with the same *CFTR* genotype who were free of liver involvement [48]. Later, a discordant liver expression was described in affected CF sib-pairs [49]. Therefore, other factors (i.e., environmental, nutritional, compliance to therapy, and genetic) modulate the liver expression in CF patients [50]. Ten years of studies supported the view that the risk for liver expression in CF patients is influenced by modifier genes. For example, we analyzed some putative modifier genes of liver cystic fibrosis phenotype like *hemochromatosis*, *transferrin receptor 2*, *ferroportin 1*, *mannose binding lectin* (MBL), *and adenosine triphosphate-binding cassette subfamily B member 4* (ABCB4) in 108 unrelated CF patients with and without liver involvement. The HYPD *MBL* defective haplotype was significantly more frequent in CF patients with a severe liver expression [51]. Furthermore, the c.834-66G>T variant of *ABCB4* was significantly less frequent in CF patients with liver disease as compared to those with no liver disease [51]. Furthermore, in a multicentric study that included the highest number of CF patients studied so far (about two thousands) we determined the prevalence of the Z and S mutations of the *A1AT* gene in two temporal cohorts of CF patients with and without liver expression. We found that Z or S mutations confer a 5-fold increased risk for severe liver disease [52]. 

### 5.3. Diagnosis

The diagnosis of CF is based on symptoms, increased sweat chloride (the gold standard for CF diagnosis), and two severe mutations in the *CFTR* disease gene [44]. *CFTR*-RD are usually associated with normal (or borderline) sweat chloride and one or both mild mutations [53], but in such patients a severe liver expression had never been described. To be noted that terms “mild” and “severe” should not refer to mutations but to phenotypes. However, such terms are commonly used to indicate *CFTR* mutations found in CF patients with pancreatic sufficiency (mild) or mutations found in CF patients with pancreatic insufficiency (severe). 

The diagnosis of liver disease in CF patients is difficult, particularly in early stages because neither laboratory nor instrumental approaches (including transient elastography, ultrasound, computed tomography, and magnetic resonance) have a satisfactory specificity. Liver biopsy is considered the gold standard by most authors, but the patchy distribution of liver alterations in CF patients reduces its sensitivity. Finally, a combination of clinical, laboratory, and instrumental data, carefully filtered by a physician experienced in CF liver diseases, is the most efficient diagnostic approach [46].

### 5.4. Genetics

The search of *CFTR* mutations is one of the most diffuse molecular analysis worldwide, because it is used to confirm diagnosis and to identify asymptomatic carriers and for prenatal [54] or preimplantation [55] diagnosis. Different techniques are available to study the 2000 mutations identified in the *CFTR* gene so far (http://www.genet.sickkids.on.ca/). About 80% alleles from CF patients are identified if panels of the most frequent mutations are tested [56]; the analysis of mutations peculiar to specific ethnic-geographic groups may increase the detection rate [57], and the scanning of *CFTR* coding regions reveals mutations in up to 90% alleles [58, 59]. Large gene rearrangements are present in about 2-3% of alleles from CF patients [60]. Finally, pathogenic mutations in noncoding region of the *CFTR* gene have been described [61, 62], but they are not currently analysed for diagnostic purposes. The detection rate of molecular analysis is lower in *CFTR-RD* [53]. No mutations specifically associated with liver disease (or to its absence) have been described so far.

## Figures and Tables

**Table 1:** Types of hereditary hemochromatosis (HH) and genes involved.

Type of HH	Inheritance	Gene	Protein	Onset and phenotype
Type 1	AR	*HFE*	HFE	Adult, moderate
Type 2 A	AR	*HJV*	Hemojuvelin	Child, severe
Type 2 B	AR	*HAMP*	Hepcidin	Child, severe
Type 3	AR	*TFR2*	Transferrin receptor	Young adult, moderate
Type 4	AD	*SLC40A1*	Ferroportin	Adult, moderate

AR: autosomal recessive; AD: autosomal dominant.
